# Hypertension prevention attitudes and propensity to achieve a healthy lifestyle in young and middle-aged adults at increased risk: a cross-sectional study

**DOI:** 10.3389/fpubh.2026.1946451

**Published:** 2026-09-10

**Authors:** Gürkan Özden, Fatma Tubay, Zeynep Karadağ Canlı

**Affiliations:** 1 Faculty of Nursing, Internal Medicine Nursing, İnönü University, Malatya, Türkiye; 2 Corpus AR, Malatya Teknokent, Malatya, Türkiye; 3 Institute of Health Sciences, Internal Medicine Nursing, İnönü University, Malatya, Türkiye

**Keywords:** chronic disease, health attitudes, healthy lifestyle, hypertension, nursing, primary prevention

## Abstract

**Introduction:**

H Hypertension is the most significant modifiable risk factor for cardiovascular disease. This study aimed to determine attitudes toward hypertension prevention, examine their relationship with the propensity to achieve a healthy lifestyle, and identify sociodemographic and health-related predictors in young and middle-aged adults.

**Methods:**

This descriptive, cross-sectional study included 351 adults without diagnosed hypertension who had at least one chronic condition associated with increased hypertension risk. Data were collected via an online survey between February and August 2025 using a validated self-report survey comprising the Demographic Information Form, the Attitudes Scale towards Prevention of Hypertension, and the Propensity to Achieve Healthy Lifestyle Scale. Descriptive statistics, Pearson correlation, and hierarchical multiple linear regression were used to analyze relationships and correlates, with significance set at *p* < 0.05.

**Results:**

The mean age was 30.73 ± 11.20 years; 67.8% were female. The mean Attitudes Scale towards Prevention of Hypertension score was 106.37 ± 12.31 (range 26–130), indicating a positive attitude, while the mean Propensity to Achieve Healthy Lifestyle scale score was 18.93 ± 4.96, a moderate level. A significant, low-level positive correlation was found between the two scales (*r* = 0.186; *p* < 0.001). Three variables were independently associated with preventive attitudes, explaining 17.5% of the variance (all *p* < 0.05): elementary education (*β* = −0.280), no previous hypertension education (*β* = −0.175), and higher propensity to achieve a healthy lifestyle (*β* = 0.153). The propensity to achieve a healthy lifestyle accounted for a small but significant increment over sociodemographic and health variables alone (Δ*R*^2^ = 0.022, *p* = 0.003).

**Conclusion:**

Although preventive attitudes were generally positive in this at-risk, normotensive sample, perceived readiness and self-efficacy for lifestyle change remained only moderate, indicating a discordance between attitude and readiness rather than a demonstrated attitude–behavior gap, since behavior itself was not measured. Educational level and structured hypertension education showed the strongest associations with preventive attitudes, underscoring the importance of early preventive interventions prioritizing groups with lower educational levels. As the design was cross-sectional, these associations should not be interpreted causally.

## 1 Introduction

Cardiovascular diseases remain the leading cause of death worldwide, and hypertension is recognized as the most important modifiable risk factor for these diseases; two-thirds of the approximately 1.3 billion people affected by this condition live in low- and middle-income countries (1). A global cohort study has shown that modifiable risk factors, including systolic blood pressure, account for more than half of all cases of cardiovascular disease and that blood pressure offers the highest prevention potential in terms of prevention (2). It has also been reported that primary preventive measures, such as lifestyle changes, reduce age-standardized rates of cardiovascular mortality (3).

This picture places individual behaviors at the center of hypertension prevention. It has been shown that adults who adhere to a healthy diet and regular physical activity have lower rates of cardiovascular morbidity and mortality (4), that a healthy lifestyle mediates the relationship between socioeconomic status and hypertension, and that healthier behaviors are associated with a lower risk of hypertension (5). Therefore, individuals’ inclination toward a healthy lifestyle forms the behavioral foundation of prevention.

While attitudes emerge as a key determinant in the adoption of preventive behaviors, there is also evidence that a positive attitude alone does not translate into preventive behavior (6, 7). For this reason, determining the level of attitudes toward hypertension prevention and the extent to which these attitudes align with a propensity to achieve a healthy lifestyle is important for the design of preventive interventions. A positive association has been reported between health literacy and healthy lifestyle behaviors (8); furthermore, it has been demonstrated that educational level is consistently associated with hypertension awareness and control (9, 10).

However, despite hypertension being an increasingly prevalent problem among young adults and the protective role played by physical activity and dietary changes in this group, the need for awareness and educational initiatives targeting young people continues to be emphasized (11). This period presents a favorable window of opportunity for early interventions aimed at primary prevention.

Lifestyle counseling, blood pressure assessment, and patient education are core components of nursing practice in hypertension care. A middle-range nursing theory developed for this field places the patient’s attitudes and beliefs about health, together with shared decision-making between nurse and patient, at the center of the therapeutic encounter (12). Nurse-managed hypertension care in primary settings has been associated with more frequent lifestyle counseling (13), and general practice nurses identify patients’ readiness and willingness to prioritize lifestyle change as the principal determinant of whether such conversations can be initiated at all (14). Assessing preventive attitudes and behavioral propensity is therefore not merely descriptive: it identifies the point at which nursing intervention should begin. This is particularly relevant to internal medicine nursing, where nurses encounter adults who are not yet hypertensive but who carry accumulating cardiometabolic risk, and where primary prevention can be embedded within routine care for other chronic conditions. Adults with established cardiometabolic, renal, endocrine, or sleep-related conditions are a particularly relevant group. They are already in regular contact with health services, yet primary prevention of hypertension is rarely a formal part of their follow-up, which makes them both a high-yield and an accessible target for nurse-led intervention.

In the current literature, the level of attitudes toward hypertension prevention, the relationship between these attitudes and the propensity to achieve a healthy lifestyle, and their sociodemographic determinants have not been comprehensively addressed, particularly among young and middle-aged adults. Among adults with at least one chronic condition associated with increased hypertension risk but no diagnosed hypertension, this study aims to (a) describe the level of attitudes toward hypertension prevention, (b) examine the relationship between these attitudes and the propensity to achieve a healthy lifestyle, and (c) identify the sociodemographic and health-related factors that predict these attitudes.

## 2 Materials and methods

### 2.1 Study design and population

This study was descriptive and cross-sectional in nature. The research was conducted online between February 2025 and August 2025. The study is reported in accordance with the STROBE (Strengthening the Reporting of Observational Studies in Epidemiology) Statement for cross-sectional studies, and a completed STROBE checklist is provided as Supplementary material. Research data were collected via an online survey created using Google Forms, distributed through social media platforms and communication tools (WhatsApp, Telegram, Facebook, Instagram, X, etc.). To reach more participants, those who were reached were asked to refer their acquaintances. Eligibility was restricted to individuals who (i) were aged 18 years or older, (ii) were literate in Turkish and resident in Türkiye, (iii) had no previously diagnosed hypertension and were not using antihypertensive medication, and (iv) had at least one chronic condition known to be associated with an increased risk of hypertension, including metabolic conditions (diabetes mellitus, obesity, and metabolic syndrome), renal conditions (chronic kidney disease and renovascular disease), endocrine conditions (thyroid disorders, primary aldosteronism, Cushing syndrome, primary hyperparathyroidism, and pheochromocytoma), or sleep-related conditions (obstructive sleep apnea), and (v) provided online informed consent. Individuals who declined consent or submitted incomplete questionnaires were not included. Because the Attitudes Scale towards Prevention of Hypertension was developed to assess attitudes toward hypertension prevention, individuals with a previously diagnosed hypertension were considered outside the scope of the study. The specified chronic conditions were recorded and included as covariates in the analytic models.

As the survey was administered anonymously online, eligibility was established by self-declaration. Age and health-related eligibility items appeared at the beginning of the questionnaire, and all eligibility and consent items were configured as mandatory fields, so that questionnaires could not be submitted without a response. Responses were restricted to one submission per Google account. No independent verification of self-reported eligibility was possible, and the resulting records were screened for completeness before analysis; the final analytic sample comprised 351 participants, whose ages ranged from 18 to 59 years. The required sample size was determined *a priori*, before data collection, using the pwr package in R via RStudio. The calculation was based on pwr.anova.test, specifying a one-way analysis of variance comparing ASPH total scores across the seven educational categories, with an effect size of *f* = 0.25 (conventionally a medium effect for analysis of variance), a two-tailed α of 0.05, and statistical power of 0.90. This yielded 41 participants per group, corresponding to a minimum of 285 in total. The final sample of 351 exceeded this requirement.

The following assessment is *post hoc* and is reported separately from the *a priori* calculation above. We note that the *a priori* specification does not correspond directly to the hierarchical regression that constitutes the study’s primary analysis. Expressed in the metric appropriate to regression, the assumed effect corresponds to *f*^2^ = 0.0625, which lies between Cohen’s conventional benchmarks for small (*f*^2^ = 0.02) and medium (*f*^2^ = 0.15) effects, and the calculation specified neither *f*^2^ nor the number of predictors ultimately entered into the model. We therefore report a *post hoc* sensitivity analysis for the achieved sample rather than restating the *a priori* calculation in terms for which it was not designed.

Accordingly, because the primary analytic model was a multiple regression containing 15 predictors, a sensitivity analysis was additionally conducted for the achieved sample. With *n* = 351, *α* = 0.05, and power of 0.80, the smallest detectable effects were *f*^2^ = 0.056 for the overall model (15 predictors, 15 and 335 df) and *f*^2^ = 0.023 for the incremental contribution of a single predictor (1 and 335 df). Both the observed model effect (*f*^2^ = 0.212) and the observed increment for the PAHLS total score (*f*^2^ = 0.027) exceeded these thresholds, indicating that the study was adequately powered for the analyses reported.

### 2.2 Data collection tools

To collect the research data, the researcher used the Demographic Information Form, the Attitudes Scale towards Prevention of Hypertension, and the Propensity to Achieve Healthy Lifestyle Scale.

Demographic Information Form: The form, prepared by the researcher, consists of 8 questions covering age, gender, marital status, income level, education level, height and weight, presence of multiple chronic conditions, and whether the respondent has received education regarding hypertension.

### 2.3 Attitudes Scale Towards Prevention of Hypertension (ASPH)

Developed by Albayrak and Şengezer (15), this scale consists of 26 items and 5 factors; items 15 and 20 are negative and are scored in reverse. The scale’s factors and items are as follows: 1. Prevention and control (items 1, 4, 7, 10, 13, 18, 22, 25); 2. Habits and lifestyle (items 6, 12, 17, 21, 24, 26); 3. Attitudes toward nutrition (questions 5, 11, 16, 20), 4. Mental state and physical activity (questions 3, 9, 15), and 5. Knowledge of disease and risk (questions 2, 8, 14, 19, 23). The scale is a 5-point Likert-type scale coded as “strongly agree (5), agree (4), undecided (3), disagree (2), and strongly disagree (1).” Scores range from 26 to 130. There is a positive correlation between hypertension prevention attitudes and scale scores. The ASPH was developed in adults who had a family member with hypertension and who did not themselves have a diagnosis of hypertension, and its developers state that it may be administered to anyone in order to assess attitudes toward hypertension prevention and used in community screening and educational settings. The Cronbach’s alpha coefficients for the ASPH, for overall reliability and subscales, are 0.91 and 0.60–0.81, respectively. In our study, the scale’s Cronbach’s alpha value was found to be 0.89.

### 2.4 Propensity to Achieve Healthy Lifestyle Scale (PAHLS)

The PAHLS was originally developed in Swedish by Broström et al. (16) to measure propensity for behavior change regarding food habits, physical activity, and weight reduction in patients with hypertension; the Turkish adaptation and psychometric evaluation were conducted by Kes et al. (17). The PAHLS is a self-report instrument consisting of 6 items. The scale was developed and published within the nursing literature, and its developers propose it as a tool that nurses can use to simplify shared decision-making regarding behavioral change.

Each domain of the scale (eating habits, physical activity, and weight loss) is based on two questions: (1) Considering your current situation, how prepared are you for X? (2) How do you assess your ability to succeed in X? The scale consists of six items. The first three items of the scale are rated as “I am very prepared, I am quite prepared, I am moderately prepared, I am somewhat prepared, I am not prepared at all,” while the last three items are rated as “I have a very high level of capacity, I have a good level of capacity, I have a moderate level of capacity, I have some capacity, I have no capacity at all.” Scores range from 6 to 30. Higher scores on the scale indicate that the patient has a greater capacity to be prepared or successful in achieving a healthy lifestyle. The PAHLS therefore captures perceived readiness and perceived capability to change rather than enacted behavior; it does not record dietary intake, physical activity, weight change, or blood pressure monitoring. Scores should accordingly be interpreted as indicators of behavioral readiness and self-efficacy.

The construct assessed by the PAHLS is not a hypertension-specific clinical characteristic. The instrument is grounded in the Transtheoretical Model and its items concern readiness and perceived capacity to change eating habits, physical activity, and weight, none of which refer to blood pressure, antihypertensive treatment, or any hypertension-specific clinical state. These constructs are meaningful irrespective of hypertension status and are of particular interest from a preventive perspective, which is why we considered the instrument conceptually appropriate for a normotensive at-risk sample.

The Cronbach’s alpha coefficient for the PAHLS scale was found to be 0.80. In our study, the Cronbach’s alpha value for the scale was found to be 0.86.

### 2.5 Data analysis

Research data were analyzed using the RStudio statistical software package. The analytic dataset contained no missing values. All eligibility, demographic, and scale items were configured as mandatory fields in the online questionnaire, so that a form could not be submitted with any item left blank, and forms that were begun but not submitted were not captured. Records were additionally screened for completeness before analysis. All analyses were therefore conducted on complete cases, and no imputation was required. Categorical variables were summarized as counts and percentages; continuous variables were summarized as mean, standard deviation, median, interquartile range, and minimum–maximum values. When comparing the total ASPH scores across groups, due to unequal group sizes, the Welch-corrected independent samples *t*-test was used for two-group comparisons, and Welch ANOVA was used for three or more groups; significant differences were further analyzed using the Games-Howell test. Effect sizes were reported as Hedges’ g for two groups and omega-squared (*ω*^2^) for multiple groups. (Results from the classical t-test and ANOVA also showed the same pattern.) Relationships with continuous variables were examined using Pearson correlation, and sensitivity to distribution was assessed using Spearman’s coefficients.

Independent predictors of the ASPH score were examined using hierarchical multiple linear regression. In the first block, gender, marital status, age, BMI, income status, education level, receipt of hypertension education, and multimorbidity (≥2 chronic conditions vs. a single condition) were included in the model; in the second block, the total PAHLS score was included. The incremental contribution of the PAHLS total score was quantified as the change in *R*^2^ from Block 1 to Block 2, tested with a nested-model F test (ΔF) and, given the use of heteroscedasticity-consistent standard errors, additionally with an HC3-based robust Wald test. Cohen’s f^2^ was computed as an effect size for the block-wise increment. Income status and educational level were coded using dummy variables; for educational level, bachelor’s degree (the modal category) served as the reference. An alternative specification treating educational level as a single ordinal trend variable was also examined; because it constrained the association to a uniform linear gradient and thereby obscured the pattern observed across categories, the categorical specification was retained. Since heteroscedasticity was detected in the White test, the coefficients were reported with robust standard errors (HC3). Multicollinearity was assessed with variance inflation factors, residual independence with the Durbin–Watson statistic, and influence with Cook’s distance and externally studentized residuals. Sensitivity analyses were conducted by refitting the final model after excluding observations with externally studentized residuals exceeding |3| and by estimating a robust regression using Tukey’s biweight estimator. All tests were two-tailed, with a significance level of *p* < 0.05 and a confidence level of 95%.

## 3 Results

A total of 351 individuals were included in the study. The participants’ mean age was 30.73 ± 11.20 years (range 18–59), and their mean BMI was 24.52 ± 4.03 kg/m^2^. 67.8% of the participants were women. 59.8% were single. 51.6% held a bachelor’s degree; 81.2% reported that they had not previously received training on hypertension. By design, all participants reported at least one chronic condition associated with increased hypertension risk; 33 participants (9.4%) reported two or more (Table 1).

**Table 1:** Distribution of participants’ demographic characteristics (*n* = 351).

Characteristic	*n*	%
Gender		
Female	238	67.8
Male	113	32.2
Marital status		
Married	141	40.2
Single	210	59.8
Income status		
Income exceeds expenses	78	22.2
Income equals expenses	163	46.4
Revenue is less than expenses	110	31.3
Educational status		
Elementary school	19	5.4
Middle school	13	3.7
High school	47	13.4
Associate’s degree	64	18.2
Bachelor’s	181	51.6
Master’s	17	4.8
Doctorate and above	10	2.8
Receiving hypertension education		
Yes	66	18.8
No	285	81.2
Multiple chronic condition		
Yes	33	9.4
No	318	90.6
Age $\bar{x}\pm s$	(30.73 ± 11.20)	
BMI $\bar{x}\pm s$	(24.52 ± 4.03)	

*n*, number of participants; BMI, body mass index; SD, standard deviation. Values are presented as *n* (%) for categorical variables and as mean ± SD for continuous variables.

While the total ASPH score (106.37 ± 12.31), concentrated in the upper part of the possible range, indicates a generally positive preventive attitude among the sample, the PAHLS mean (18.93 ± 4.96) remained at the midpoint of the range. The ASPH distribution is left-skewed (skewness = −0.79; kurtosis = 2.74); the PAHLS distribution is approximately symmetric (skewness = −0.18; kurtosis = 0.54) (Table 2).

**Table 2:** Descriptive statistics of scale and subscale scores (*n* = 351).

Scale/subscale	Mean ± SD	Min–max	Possible range	Normality
ASPH—Total	106.37 ± 12.31	54–130	26–130	Skewness = −0.79 Kurtosis = 2.74
Prevention and control	33.14 ± 3.95	16–40	8–40	
Habits and lifestyle	24.10 ± 3.41	11–30	6–30	
Dietary attitudes	16.33 ± 2.46	8–20	4–20	
Mental health and physical activity	12.68 ± 1.80	5–15	3–15	
Disease and risk awareness	20.12 ± 2.86	8–25	5–25	
PAHLS—Total	18.93 ± 4.96	6–30	6–30	Skewness = −0.18 Kurtosis = 0.54

ASPH, Attitudes Scale towards Prevention of Hypertension; PAHLS, Propensity to Achieve Healthy Lifestyle Scale; SD, standard deviation; Min, minimum; Max, maximum.

ASPH scores differed significantly by educational status [*F*(6; 52.4) = 6.40; *p* < 0.001; *ω*^2^ = 0.083]. Scores among those who received hypertension education were significantly higher than those who did not (*t*(93.3) = 3.66; *p* < 0.001; *g* = 0.52 [0.25–0.79]). No significant differences were found in terms of gender, marital status, income, or multimorbidity (Table 3). Games-Howell *post hoc* comparisons indicated that the differences by educational status were confined to contrasts between pre-tertiary and tertiary groups. Participants with middle school (mean difference = 8.00; 95% CI: 2.51–13.49; *p* = 0.002), elementary school (14.15; 95% CI: 1.29–27.00; *p* = 0.025), and high school education (6.13; 95% CI: 0.38–11.88; *p* = 0.029) scored significantly lower than bachelor’s degree holders, and elementary school graduates also scored lower than those with a doctorate (16.93; 95% CI: 0.61–33.24; *p* = 0.038). No other pairwise comparison reached significance; in particular, no two tertiary categories differed from one another.

**Table 3:** Comparison of total ASPH scores by demographic characteristics.

Variable	Category	Mean ± SD	Test	*p*	Effect
Gender	Female	106.21 ± 11.69	*t*(193.4) = −0.33	0.742	*g* = −0.04 [−0.26–0.18]
	Male	106.70 ± 13.57			
Marital status	Married	105.84 ± 13.12	*t*(277.5) = −0.65	0.518	*g* = −0.07 [−0.29–0.14]
	Single	106.72 ± 11.75			
Income status	Income exceeds expenses	107.69 ± 11.93	*F*(2; 193.2) = 0.70	0.499	*ω*^2^ = 0.000
	Income equals expenses	105.72 ± 12.41			
	Revenue is less than expenses	106.38 ± 12.47			
Educational status	Elementary school	94.47 ± 16.75	*F*(6; 52.4) = 6.40	<0.001	*ω*^2^ = 0.083
	Middle school	100.62 ± 5.30			
	High School	102.49 ± 11.69			
	Associate’s degree	105.77 ± 12.51			
	Bachelor’s	108.62 ± 11.11			
	Master’s	110.12 ± 13.78			
	Doctorate and above	111.40 ± 10.66			
Receiving hypertension education	Yes	111.47 ± 12.72	*t*(93.3) = 3.66	<0.001	*g* = 0.52 [0.25–0.79]
	No	105.19 ± 11.93			
Multiple chronic disease	Yes	109.88 ± 12.85	*t*(38.3) = 1.66	0.106	g = 0.32 [−0.04–0.67]
	No	106.00 ± 12.22			

ASPH, Attitudes Scale towards Prevention of Hypertension; SD, standard deviation; *t*, Welch-corrected independent samples *t*-test statistic; *F*, Welch analysis of variance statistic; *g*, Hedges’ *g*; *ω*^2^, omega squared; CI, confidence interval. Degrees of freedom are given in parentheses. The Welch *t*-test was used for two-group comparisons, and Welch ANOVA was used for multiple groups. Post hoc comparisons for educational status were conducted using the Games-Howell test; significant pairwise contrasts are reported in the text.

Relationships between the dependent variable and continuous variables were examined using the Pearson correlation coefficient; the Spearman correlation coefficient was also reported for validation purposes (Table 4). A positive, low-level significant relationship (*r* = 0.186; *p* < 0.001) was found between the total ASPH score and the propensity to achieve a healthy lifestyle. In contrast, no significant relationship was found with age or BMI.

**Table 4:** Correlations between the ASPH total score and continuous/ordinal variables.

Variable	Pearson *r*	*p*	Spearman *ρ*	*p*
Educational status	0.308	<0.001	0.283	<0.001
PAHLS total	0.186	<0.001	0.183	<0.001
Age	0.025	0.644	0.054	0.310
BMI	0.021	0.696	0.027	0.618
Income status	−0.032	0.544	−0.028	0.604

ASPH, Attitudes Scale towards Prevention of Hypertension; PAHLS, Propensity to Achieve Healthy Lifestyle Scale; BMI, body mass index; *r*, Pearson correlation coefficient; *ρ*, Spearman rank correlation coefficient.

Independent predictors of the ASPH total score were examined using hierarchical multiple linear regression with HC3 robust standard errors. Block 1, comprising gender, marital status, age, BMI, income level, educational level, prior hypertension education, and multimorbidity, accounted for 15.3% of the variance in preventive attitudes (*R*^2^ = 0.153, adjusted *R*^2^ = 0.118, *F*(14, 336) = 4.33, *p* < 0.001). Adding the PAHLS total score in Block 2 produced a small but statistically significant increment of 2.2 percentage points (Δ*R*^2^ = 0.022, Δ*F*(1, 335) = 9.05, *p* = 0.003; HC3 robust Wald *F*(1, 335) = 7.68, *p* = 0.006; Cohen’s *f*^2^ = 0.027; semipartial *r* = 0.149). The final model explained 17.5% of the variance in preventive attitude scores (*R*^2^ = 0.175, adjusted *R*^2^ = 0.138, *F*(15, 335) = 4.74, *p* < 0.001) (Table 5).

**Table 5:** Final multiple linear regression model for the ASPH total score.

Predictor	B	HC3	*β*	95% CI	*p*	VIF
Male (female ref.)	−2.284	1.827	−0.087	−5.877–1.309	0.212	1.32
Single (married ref.)	1.101	1.995	0.044	−2.823–5.025	0.581	2.49
Age	0.136	0.106	0.123	−0.074–0.345	0.203	2.96
BMI	0.265	0.212	0.087	−0.151–0.682	0.211	1.45
Revenue = expenses (revenue > expenses ref.)	−1.585	1.642	−0.064	−4.815–1.644	0.335	1.75
Revenue < expenses (revenue > expenses ref.)	−0.961	1.890	−0.036	−4.679–2.758	0.612	1.78
Elementary school (bachelor’s degree ref.)	−15.227	4.731	−0.280	−24.532–-5.921	0.001	1.24
Middle school	−9.047	2.320	−0.139	−13.610–-4.484	<0.001	1.15
High school	−5.280	1.968	−0.146	−9.151–-1.408	0.008	1.20
Associate’s degree	−2.267	1.806	−0.071	−5.819–1.284	0.210	1.16
Master’s degree	−0.936	3.643	−0.016	−8.102–6.230	0.797	1.18
Doctorate degree	0.866	4.397	0.012	−7.784–9.515	0.844	1.18
No HT training (with training ref.)	−5.490	1.777	−0.175	−8.986–-1.995	0.002	1.09
Multiple chronic disease (no ref.)	3.038	2.412	0.072	−1.707–7.782	0.209	1.07
PAHLS total score	0.381	0.138	0.153	0.111–0.652	0.006	1.06

ASPH, Attitudes Scale towards Prevention of Hypertension; PAHLS, Propensity to Achieve Healthy Lifestyle Scale; BMI, body mass index; B, unstandardized regression coefficient; HC3, heteroscedasticity-consistent (HC3) robust standard error; *β*, standardized regression coefficient; CI, confidence interval; VIF, variance inflation factor; *R*^2^, coefficient of determination; Δ*R*^2^, change in *R*^2^; ref., reference category. Block 1 (sociodemographic and health variables): *R*^2^ = 0.153, adjusted *R*^2^ = 0.118, *F*(14, 336) = 4.33, *p* < 0.001. Block 2 (+ PAHLS total score): *R*^2^ = 0.175, adjusted *R*^2^ = 0.138, *F*(15, 335) = 4.74, *p* < 0.001; Δ*R*^2^ = 0.022, Δ*F*(1, 335) = 9.05, *p* = 0.003. Coefficients shown are from the final (Block 2) model.

Educational level was the strongest correlate of preventive attitudes. Compared with participants holding a bachelor’s degree, those with an elementary school education scored 15.227 points lower (B = −15.227, HC3 SE = 4.731, *β* = −0.280, 95% CI: −24.532 to −5.921, *p* = 0.001), middle school graduates 9.047 points lower (B = −9.047, HC3 SE = 2.320, *β* = −0.139, 95% CI: −13.610 to −4.484, *p* < 0.001), and high school graduates 5.280 points lower (B = −5.280, HC3 SE = 1.968, *β* = −0.146, 95% CI: −9.151 to −1.408, *p* = 0.008). Scores of participants with associate (B = −2.267, *p* = 0.210), master’s (B = −0.936, *p* = 0.797), or doctoral (B = 0.866, *p* = 0.844) degrees did not differ significantly from those of bachelor’s degree holders.

Participants who had not previously received hypertension education scored 5.490 points lower than those who had (B = −5.490, HC3 SE = 1.777, *β* = −0.175, 95% CI: −8.986 to −1.995, *p* = 0.002). The PAHLS total score was also independently and positively associated with the ASPH score, with each one-point increase corresponding to a 0.381-point increase in preventive attitudes (B = 0.381, HC3 SE = 0.138, *β* = 0.153, 95% CI: 0.111–0.652, *p* = 0.006).

Gender, marital status, age, body mass index, income level, and the presence of multimorbidity were not significantly associated with the ASPH total score (all *p* > 0.05). Variance inflation factors ranged from 1.06 to 2.96, indicating no substantial multicollinearity in the final model (Table 5).

Regression diagnostics indicated no undue influence: the maximum Cook’s distance was 0.116, well below the conventional threshold of 1, the Durbin–Watson statistic was 1.96, and variance inflation factors ranged from 1.06 to 2.96. Six observations exhibited externally studentized residuals exceeding |3|. In a sensitivity analysis excluding these observations, the direction and significance of every predictor in the final model were preserved (elementary school B = −10.771, *p* < 0.001; middle school B = −9.356, *p* < 0.001; high school B = −4.625, *p* = 0.003; no prior hypertension education B = −5.636, *p* < 0.001; PAHLS B = 0.307, *p* = 0.015; *R*^2^ = 0.176). A robust regression using Tukey’s biweight estimator yielded the same pattern for the principal predictors (elementary school B = −8.296, *p* = 0.002; middle school B = −8.618, *p* = 0.005; no prior hypertension education B = −6.123, *p* < 0.001; PAHLS B = 0.341, *p* = 0.003), although the high school contrast fell short of significance under this estimator (B = −3.258, *p* = 0.062). Two departures from the primary model warrant note: the elementary school coefficient attenuated substantially under both procedures, indicating that its magnitude in the primary model is partly carried by a small number of low-scoring observations within a category of only 19 participants; and the coefficient for multiple chronic conditions, non-significant in the primary model, reached significance when outlying observations were excluded (B = 4.001, *p* = 0.019) but is reported here only as a sensitivity result and is not interpreted further.

## 4 Discussion

This study examined the level of hypertension prevention attitudes among 351 individuals aged 18–59 who had at least one chronic condition associated with increased hypertension risk but no diagnosed hypertension. The sample’s total score on the ASPH (106.37 ± 12.31) was concentrated in the upper range of the possible score range (26–130); and the left-skewed distribution (skewness = −0.79) indicated that the vast majority of participants exhibited a relatively positive prevention attitude. In contrast, the mean score on the PAHLS (18.93 ± 4.96) remained at the midpoint of the possible range. In the multivariate analysis, educational level, prior hypertension education, and the propensity to achieve a healthy lifestyle were independently associated with preventive attitudes; no significant association was found with gender, marital status, income, multiple chronic conditions, age, or body mass index (BMI).

The fact that the sample generally demonstrated a positive attitude toward prevention is consistent with the findings of knowledge–attitude–behavior (KAB) studies conducted in various societies. It has been reported that 77.3% of participants among children and adolescents in Nigeria exhibited a positive attitude toward prevention (18), that this rate was 63.8% among adolescents in Indonesia (6), and that the general population in Saudi Arabia possesses a good level of knowledge and positive beliefs regarding hypertension prevention (19). However, there are also studies pointing out that a positive attitude alone does not translate into preventive behavior; it has been shown that a positive attitude does not guarantee preventive behavior among adolescents (6) and that knowledge alone does not predict behavioral change among young adults (7). Indeed, in Namibia, although the majority of participants among health sciences students and staff considered hypertension to be a serious problem, only about 39% had their blood pressure measured regularly, and only half were implementing changes in physical activity and diet (20). Our own findings, however, require a more precise characterization than this literature permits. The studies cited above measured enacted behavior, whereas the PAHLS assesses how prepared individuals feel and how capable they judge themselves to be of changing diet, physical activity, and weight. What we observed is therefore not an attitude–behavior gap in the strict sense, since no behavioral outcome was recorded, but a discordance between high preventive attitudes and only moderate perceived readiness and self-efficacy, which we refer to hereafter as an attitude–readiness gap. This distinction matters theoretically: an attitude–behavior gap may arise from environmental or structural barriers that intervene between intention and action, whereas the pattern observed here locates the constraint earlier in the causal chain, at the point where individuals appraise their own capacity to act. It would be consistent with the transtheoretical proposition that self-efficacy governs progression from contemplation to preparation and action, although our cross-sectional design cannot establish that readiness constrains preventive action, nor rule out the reverse ordering in which prior behavioral experience shapes both attitudes and perceived capability. Establishing which construct is limiting would require longitudinal or experimental designs. For nursing practice, this gap locates the point at which intervention is required. Since attitudes were already favourable in this sample while perceived readiness was not, behavior-oriented approaches that can be embedded in routine nursing encounters, such as motivational interviewing and structured goal-setting, may be a more promising avenue than further information provision alone (21). Whether such approaches produce better outcomes than education focused on attitudes remains an empirical question that our data cannot answer.

A notable feature of our findings is that the association between educational level and preventive attitudes did not appear to follow a uniform gradient across all educational categories. With bachelor’s degree holders as the reference category, scores were significantly lower among elementary school (B = −15.227; *β* = −0.280; *p* = 0.001), middle school (B = −9.047; *β* = −0.139; *p* < 0.001), and high school graduates (B = −5.280; *β* = −0.146; *p* = 0.008), whereas associate’s, master’s, and doctoral levels did not differ significantly from the bachelor’s level (all *p* > 0.05). Descriptively, the point estimates narrowed across the pre-tertiary categories (−15.2, −9.0, and −5.3 points, respectively) while the tertiary categories did not differ detectably from one another. We would emphasise, however, that these adjacent categories were not formally distinguishable from each other, and that several cells were very sparsely populated, containing as few as 10 to 19 participants, with correspondingly wide confidence intervals. The pattern is therefore best described as a tendency for differences to be concentrated below the tertiary level in this sample, and we do not regard it as establishing a threshold at any particular educational transition. In the primary model, the deficit observed among elementary school graduates (relative to university graduates) exceeded one standard deviation of the scale (SD = 12.31), indicating that this difference was meaningful in practical as well as statistical terms. Although the coefficient attenuated somewhat in sensitivity analyses (ranging from 8 to 11 points across specifications), the deficit remained substantial and statistically significant under every specification examined; the exact magnitude should therefore be regarded as approximate. Based on the standardized coefficients, having only an elementary education showed the strongest association in the model (*β* = −0.280). This pattern is consistent with evidence that educational level is positively associated with hypertension awareness and control (9, 10) and that knowledge is significantly related to education among older adults with hypertension (22). The demonstration that healthy lifestyle behaviors partially mediate the association between socioeconomic status and hypertension (5) further suggests that education operates through health literacy and behavioral resources. Population-level evidence is consistent with this mechanism: in a cohort of 8,213 adults in Tehran, lower educational attainment was independently associated with insufficient physical activity, alongside older age, unemployment, higher BMI, and the presence of diabetes, hypertension, or coronary artery disease (23). Educational disadvantage therefore appears to shape not only preventive attitudes, as observed here, but also the enacted behaviors through which those attitudes would have to operate. Clinically, this pattern suggests that adults without tertiary education may warrant particular attention in nurse-led prevention. For adults who have not entered higher education, literacy-sensitive strategies such as plain language, visual materials, and teach-back to verify understanding are indicated in preference to standard written leaflets (24). This distinction matters most in internal medicine settings, where such materials are routinely distributed without any assessment of comprehension. If this pattern were confirmed in samples recruited by probability methods and with adequate representation of each educational category, its implications would extend beyond the clinical encounter, since adults with less formal education may not be reached through clinical channels alone. Our data are not sufficient to establish such a claim, but they are consistent with the broader literature on educational differences in hypertension awareness cited above, and they suggest that prevention messaging delivered through school, workplace, and community settings merits evaluation alongside clinically delivered education.

Participants who had previously received hypertension education scored higher than those who had not, with a moderate effect size (*g* = 0.52). In the multivariate model, the absence of such education was associated with an adjusted score 5.49 points lower (*β* = −0.175; *p* = 0.002), indicating an independent contribution. This finding is supported by experimental studies demonstrating that structured health education initiatives improve attitudes toward hypertension. It has been shown that multimedia-based education significantly increases attitude scores in patients with hypertension (25) and that presentation, and video-based health education positively enhances preventive attitudes in at-risk individuals (26). The fact that only 18.8% of participants in our sample reported having previously received hypertension education points to a significant gap in this area; when evaluated alongside recommendations for the widespread implementation of school- and community-based hypertension education programs (18). Since structured education of this kind falls squarely within the nursing remit in both hospital and community settings, the finding that fewer than one in five participants had ever received it identifies an unmet need that nurses are directly positioned to address.

A positive but weakly significant relationship was found between the propensity to achieve a healthy lifestyle and preventive attitudes (*r* = 0.186); in the multivariate model, this variable remained independently associated with the outcome (B = 0.381; *β* = 0.153; *p* = 0.006). In hierarchical terms, however, the propensity to achieve a healthy lifestyle added only 2.2 percentage points to the variance explained by sociodemographic and health variables alone (Δ*R*^2^ = 0.022, *f*^2^ = 0.027), a small increment that quantifies rather than contradicts the attitude–readiness gap outlined above: propensity to achieve a healthy lifestyle is reliably but only marginally informative about preventive attitudes once education and prior hypertension education are accounted for. A one-unit increase in the PAHLS score corresponds to an average increase of 0.38 points in the preventive attitude score, holding all other variables constant. The direction of the relationship is as expected: findings from a meta-analysis reporting a positive but weak relationship between health literacy and healthy lifestyle behaviors (8) and a study showing that healthy lifestyle behaviors predict health-promoting attitudes (27) support this pattern. Findings from a study in Turkey suggesting that systemic blood pressure can be reduced by improving lifestyle among university students (28) also highlight the clinical importance of the link between attitude and perceived readiness to change. Larger-scale evidence points in the same direction: among 9,388 hypertensive adults in a nationwide Iranian survey, adherence to a composite healthy lifestyle was associated with modest but statistically significant reductions in systolic and diastolic blood pressure, independent of antihypertensive medication use, although the association with hypertension control itself did not reach significance (29). We note that this study, like the others cited here, assessed enacted lifestyle behavior rather than readiness to change; it therefore establishes the clinical value of the behavioral endpoint toward which readiness is a precursor, rather than validating readiness as a proxy for it. The fact that the correlation remains at a low level suggests that preventive attitudes and perceived readiness are interconnected yet distinct constructs; a positive preventive attitude is reflected only partially in perceived readiness and self-efficacy. This distinction has practical consequences for assessment. Because attitudes and behavioral propensity are separable, favourable attitudes cannot be taken as evidence of readiness to act. Brief assessment of both during routine encounters would allow nurses to identify individuals whose positive attitudes are not matched by behavioral capacity and to direct counseling accordingly, consistent with the emphasis of nursing theory in hypertension care on supporting self-care agency rather than transmitting knowledge (12).

The absence of significant associations for gender, marital status, income, multimorbidity, age, and BMI warrants careful interpretation, and we regard these as inconclusive rather than as evidence of no association. Contrary to meta-analytic evidence that health literacy is higher among women (8), attitude scores did not differ by gender in our sample; and contrary to studies linking socioeconomic status to blood-pressure control behavior (9, 30), income was not significantly associated with preventive attitudes. The most plausible explanation lies in the composition of the sample rather than in the absence of true effects. Age was strongly clustered in early adulthood (median 26 years, interquartile range 22–39; 61.5% were aged 30 years or younger and only 9.7% were aged 50 or above), and BMI was similarly concentrated within the normal range (54.1% normal weight; only 10.3% obese). Because the sample contained few participants in the age and BMI ranges where hypertension risk is most concentrated, the statistical power available to detect such associations was substantially reduced. Restriction of range attenuates observed associations irrespective of their true magnitude, and our null findings for age and BMI should therefore be read as a property of this sample rather than as a general result (31).

The null finding for income requires a different explanation, since income was in fact distributed across all three categories (22.2, 46.4, and 31.3%). Here the more likely limitation is measurement: income was assessed with a single, three-level subjective item comparing income with expenses, which captures perceived economic adequacy only coarsely and is subject to considerable measurement error. Such coarse categorisation attenuates associations and may have obscured a relationship that a continuous or multi-indicator measure of socioeconomic position would have detected (32). A second consideration concerns what this item is likely to have measured in a sample of this composition. Almost half of the participants were aged 25 years or younger (49.0%), and the great majority of these were unmarried, so that a substantial proportion of the sample was probably of student age and economically dependent rather than in employment. For such respondents, a judgement of whether income meets expenses reflects the adequacy of parental or household support and the circumstances of student life rather than their own position in the socioeconomic structure. The distribution is consistent with this reading: participants aged 25 or younger were considerably more likely than older participants to report that income fell short of expenses (39.0% versus 24.0%). Subjective income adequacy is therefore a poor proxy for socioeconomic standing in this sample, and its inclusion cannot be taken to have tested the socioeconomic hypothesis adequately. Our data consequently permit no conclusion about socioeconomic gradients in preventive attitudes beyond those already captured by educational level, and studies employing occupational classification, household income, or composite indices of socioeconomic position are needed to establish whether such gradients exist (33). Similarly, the comparison by presence of multiple chronic conditions rested on only 33 affected participants, and the observed difference of nearly four points (*g* = 0.32), while not statistically significant, is not negligible in magnitude. We therefore refrain from concluding that these characteristics are unrelated to preventive attitudes, and we suggest that adequately powered studies with broader age, BMI, and socioeconomic distributions are needed before such a conclusion could be drawn.

The fact that the variance explained by the final model remains at a modest level (*R*^2^ = 0.175) reflects the multifactorial nature of preventive attitudes. Beyond the sociodemographic and health variables included in the model, it is known that factors such as risk perception, family history, and health literacy play a role in shaping attitudes; indeed, it has been shown that high risk perception is associated with protective behavior (18), and that health literacy and cultural factors influence preventive attitudes among young adults (7). Including these variables in future models may increase the explained variance.

### 4.1 Limitations

Several limitations of this study should be acknowledged. First, the cross-sectional design precludes causal interpretation; the direction of the relationship between educational level and attitudes, as well as possible mediating mechanisms, requires examination in longitudinal designs.

Second, convenience and snowball sampling through social media without a defined sampling frame limited the representativeness and generalizability of the findings: a response rate could not be calculated, self-selection bias cannot be excluded, and whether non-completion was systematically related to the variables of interest could not be examined because unsubmitted questionnaires were not recorded. Health-engaged individuals may have been more likely to participate, which could explain the high, left-skewed ASPH scores and the possible ceiling effect; the observed attitude level should therefore be regarded as an upper-bound estimate, although the low proportion of participants reporting prior hypertension education (18.8%) indicates that the sample was not composed exclusively of individuals already engaged with this topic. The sample was also predominantly female (67.8%), highly educated (59.3% with at least a bachelor’s degree), relatively young, and largely within the normal BMI range. This composition narrowed the observed distributions of age and BMI, weakening the associations that could be detected and further limiting generalizability to older and more heterogeneous populations. Moreover, eligibility was restricted to adults with at least one chronic condition associated with increased hypertension risk, so the findings characterize a group already at elevated risk and should not be extrapolated to healthy adults. Replication using population-based probability sampling is therefore warranted.

Third, no behavioral outcome was measured. The PAHLS records perceived readiness and capability rather than enacted diet, physical activity, weight change, or blood pressure monitoring, so whether participants’ actual preventive behavior lags behind their attitudes cannot be established; studies pairing attitude measures with objective or diary-based behavioral indicators are required.

Fourth, all data were self-reported. Social desirability may have influenced responses, and the anonymous online format precluded verification of self-declared eligibility, age, or health status. Completing both scales in a single session also raises the possibility of common-method variance; however, the observed association was small (*r* = 0.186; Δ*R*^2^ = 0.022), and the variables showing the strongest associations were single demographic items not subject to shared method effects, suggesting that any inflation was limited. Even so, the attitude–readiness association should be regarded as an upper bound.

Fifth, the two instruments differ in how closely their validation samples match ours. The ASPH was developed in adults without a hypertension diagnosis, although that study excluded participants with diabetes and selected on family history of hypertension. The Turkish PAHLS was validated in 132 hypertensive outpatients who differed from our participants in BMI, blood pressure, and education, and its psychometric properties have not been established in normotensive populations. Internal consistency was high in the present sample (*α* = 0.89 and 0.86), but formal psychometric evaluation in normotensive adults with chronic conditions would be worthwhile. Socioeconomic position was likewise captured only by a single three-level subjective item, which in a sample of this age composition reflects household support as much as the respondent’s own standing.

## 5 Conclusion

This study showed that attitudes toward hypertension prevention were generally positive among young and middle-aged adults with at least one chronic condition associated with increased hypertension risk. Educational attainment, previous hypertension education, and the propensity to achieve a healthy lifestyle were independently associated with preventive attitudes. These findings highlight the need for literacy-sensitive and behavior-oriented educational interventions, particularly for adults who have not entered higher education and those who have not previously received hypertension education. However, given the cross-sectional design and a self-selected convenience sample that was young, predominantly female, and highly educated, these findings describe the population studied rather than adults in general, and the non-significant results for age, BMI, and income should be regarded as inconclusive rather than as evidence of no association. Because nurses play a central role in delivering lifestyle counseling across healthcare settings, these findings have important implications for nurse-led primary prevention. In particular, they support the routine assessment of preventive attitudes, the provision of education tailored to individuals’ educational and health literacy levels, and behavior-oriented counseling aimed at strengthening perceived readiness and self-efficacy, which were only moderate in this sample even where preventive attitudes were favourable. Clinical channels alone, however, will not reach those most in need. Because the educational differences observed here were most apparent below the tertiary level, though not precisely delineated in a sample of this composition, extending literacy-sensitive prevention beyond healthcare encounters into school-based, workplace, and community programs merits consideration and evaluation. Longitudinal and intervention studies are needed to determine whether improvements in preventive attitudes lead to sustained changes in lifestyle behaviors and hypertension risk.

## Data Availability

The raw data supporting the conclusions of this article will be made available by the authors, without undue reservation.
